# Genomics of Methylotrophy in Gram-Positive Methylamine-Utilizing Bacteria

**DOI:** 10.3390/microorganisms3010094

**Published:** 2015-03-20

**Authors:** Tami L. McTaggart, David A. C. Beck, Usanisa Setboonsarng, Nicole Shapiro, Tanja Woyke, Mary E. Lidstrom, Marina G. Kalyuzhnaya, Ludmila Chistoserdova

**Affiliations:** 1 Department of Chemical Engineering, University of Washington, Seattle, WA 98195, USA; E-Mails: tmctagga@uci.edu (T.L.M.); dacb@uw.edu (D.A.C.B.); lisa.setboonsarng@gmail.com (U.S.); lidstrom@uw.edu (M.E.L.); 2 Department of Microbiology, University of Washington, Seattle, WA 98195, USA; E-Mail: mkalyuzhnaya@mail.sdsu.edu; 3 eScience Institute, University of Washington, Seattle, WA 98195, USA; 4 DOE Joint Genome Institute, Walnut Creek, CA 94598, USA; E-Mails: nrshapiro@lbl.gov (N.S.); TWoyke@lbl.gov (T.W.)

**Keywords:** methylotrophy, genomics, *Arthrobacter*, *Bacillus*, *Mycobacterium*, Lake Washington

## Abstract

Gram-positive methylotrophic bacteria have been known for a long period of time, some serving as model organisms for characterizing the specific details of methylotrophy pathways/enzymes within this group. However, genome-based knowledge of methylotrophy within this group has been so far limited to a single species, *Bacillus methanolicus* (Firmicutes). The paucity of whole-genome data for Gram-positive methylotrophs limits our global understanding of methylotrophy within this group, including their roles in specific biogeochemical cycles, as well as their biotechnological potential. Here, we describe the isolation of seven novel strains of Gram-positive methylotrophs that include two strains of *Bacillus* and five representatives of Actinobacteria classified within two genera, *Arthrobacter* and *Mycobacterium*. We report whole-genome sequences for these isolates and present comparative analysis of the methylotrophy functional modules within these genomes. The genomic sequences of these seven novel organisms, all capable of growth on methylated amines, present an important reference dataset for understanding the genomic basis of methylotrophy in Gram-positive methylotrophic bacteria. This study is a major contribution to the field of methylotrophy, aimed at closing the gap in the genomic knowledge of methylotrophy within this diverse group of bacteria.

## 1. Introduction

Methylotrophy, the ability to utilize substrates containing no carbon-carbon bonds (C1 substrates), is widespread in the domain of Bacteria, with representatives found in Proteobacteria, Firmicutes, Actinobacteria, Verrucomicrobia and the NC10 candidate phylum [1,2]. At the genomic level, methylotrophy has been characterized in detail in Proteobacteria, with the first genome published in 2004 [3] and the count of currently available genomes nearing one hundred [2]. While methylotroph representatives within Verrucomicrobia and the NC10 candidate phylum have only been characterized recently, representative genome sequences are already available [4,5,6].

Gram-positive methylotrophs have been known for a long time, some serving as model organisms for characterizing the specific details of methylotrophy pathways/enzymes within this group [1,7,8,9,10,11,12]. However, whole-genome knowledge of methylotrophy in Gram-positive methylotrophs has been limited to a single species, *Bacillus methanolicus*, within the Firmicutes [13,14,15]. Methylotrophy pathways have been analyzed in this organism at the systems level [15]. In addition, a large body of research exists on individual enzymes/pathways in a variety of Gram-positive methylotrophs, providing helpful clues as to the important methylotrophy activities in these organisms [1,7,8,9,10,11,12,15,16,17,18]. Based on the prior research, the methylotrophy metabolic scheme in Gram-positive bacteria is as follows. Primary C1 substrates, methanol or methylated amines, are oxidized to produce formaldehyde, which is assimilated via the ribulose monophosphate (RuMP) cycle into biomass (fructose 1,6-bisphosphate aldolase variant [1,15]. The oxidative branch of the RuMP cycle serves to oxidize formaldehyde to CO_2_. However, a linear route with formate as an intermediate may also be functional. In *B. methanolicus*, the linear route involves tetrahydrofolate (H_4_F)-linked C1 transfer reactions [15].

The goal of this study was to expand the whole-genome knowledge of methylotrophy in Gram-positive bacteria through comparative genomics of divergent taxa within this group. Here, we describe the isolation of seven novel strains of Gram-positive methylotrophs, including two strains of *Bacillus* (*Bacillaceae*) and five representatives of Actinobacteria, belonging to the genera of *Arthrobacter* (*Micrococcaceae*) and *Mycobacterium* (*Microbacteriaceae*). We report whole-genome sequences for these isolates and present a comparative analysis of methylotrophy functional modules within these genomes.

## 2. Materials and Methods

### 2.1. Permissions

No specific permissions were required for the sampling location (47° 38.075′ N, 122° 15.993′ W) or sampling activities. The field studies did not involve endangered or protected species.

### 2.2. Strain Isolation and Cultivation

*Arthrobacter* sp. MA-N2 was isolated from a 2004 Lake Washington sample [19], and the rest of the strains were isolated from a 2011 Lake Washington sample ([20]; Table 1). All strains were isolated from methylamine enrichments, which were set up as previously described [20,21]. Axenic cultures were routinely maintained on solid media supplemented by methylamine. For long-term storage, culture stocks were frozen at −80° with 10% dimethyl sulfoxide, as a cryoprotective agent.

### 2.3. DNA Isolation, Whole Genome Sequencing, Assembly and Genome Annotation

Biomass for genomic DNA isolation was collected from plates. DNA was isolated as previously described [22]. The draft genomes were generated at the Department of Energy Joint genome Institute (JGI) using Illumina, Pacific Biosciences (PacBio) or a combination of the two technologies (see Table 1). All general aspects of library construction and sequencing performed at the JGI can be found at http://www.jgi.doe.gov. The raw reads were assembled using HGAP (version: 2.0.0) [23] for PacBio datasets, Allpaths, Version r41554, for hybrid Illumina/PacBio datasets, and a combination of Allpaths, Version r41554 [24], and Velvet, Version 1.1.05 [25], for the Illumina datasets. Genes were identified using Prodigal [26], followed by a round of manual curation using GenePRIMP [27] for the Draft genomes in fewer than 10 scaffolds. The predicted coding sequences were translated and used to search the National Center for Biotechnology Information (NCBI) non-redundant database, Universal Protein Resource (UniProt), TIGRFam, Pfam, Kyoto Encyclopedia of Genes and Genomes (KEGG), COG, and InterPro databases. The tRNAScanSE tool [28] was used to find tRNA genes, whereas ribosomal RNA genes were found by searches against models of the ribosomal RNA genes built from SILVA [29]. Other non-coding RNAs, such as the RNA components of the protein secretion complex and the RNase P, were identified by searching the genome for the corresponding Rfam profiles using INFERence of RNA Alignment (INFERNAL) [30]. Additional gene prediction analysis and manual functional annotation was performed within the Integrated Microbial Genomes (IMG) platform developed by the Joint Genome Institute, Walnut Creek, CA, USA [31].

The genomes are accessible through the IMG interface (http://img.jgi.doe.gov/cgi-bin/w/main.cgi).

**Table 1:** Strain isolation details, genome statistics and accession numbers.

Strain	Year Isolated	Enrichment Temperature °C	Total Nucleotides	GC%	Sequencing Technology	Scaffolds	Coverage (X)	NCBI Accession Number
*Arthrobacter* sp. 31Y	2011	10	5,079,550	61.94	PacBio	2	140	JAFW00000000.1
*Arthrobacter* sp. 35W	2011	10	4,660,196	66.71	Illumina/PacBio	4	1647/130	AXVQ00000000.1
*Arthrobacter* sp. MA-N2	2004	Room	4,833,792	62.96	Illumina	5	793	AQRI01000000.1
*Bacillus* sp. 37MA	2011	10	3,981,584	40.52	Illumina	5	1091	ARCN01000000.1
*Bacillus* sp. 72	2011	10	3,908,751	40.85	PacBio	26	180	JQMI01000000.1
*Mycobacterium* sp. 141	2011	30	4,544,736	65.57	Illumina	3	1171	ARNS01000000.1
*Mycobacterium* sp. 155	2011	30	4,609,894	65.62	Illumina	2	1451	AREU01000000.1

### 2.4. Phylogenetic Analysis

Average amino acid identity (AAI) values were computed via reciprocal BLAST best hits between pairs of proteomes in accordance with previously described methods [32], except that the predicted protein products were used directly as the subject for alignments rather than translated genomic sequences [20,21].

### 2.5. Reconstruction of Methylotrophy Pathways

Automated gene annotations created using the IMG pipeline were curated manually for genes involved in key metabolic pathways. Reconstruction of methylotrophy pathways was modeled after prior analysis of the genomes of *B. methanolicus* [13,14,15]. Proteins with experimentally proven functions in methylated amine metabolism were also employed [10,18]. In the case of multiple functional counterparts, these were categorized into “types” based on reciprocal BLAST comparisons. In the cases of very divergent counterparts, some of the “type” categories are very tentative, as low homology may result either from a long evolutionary history or from a lateral transfer from a distant source.

## 3. Results and Discussion

### 3.1. Gram-Positive Methylotrophs Isolated from Lake Washington Are All Facultative Methylated Amine Utilizers

As part of a large-scale methylotroph isolation project from Lake Washington sediment samples, a number of Gram-positive strains were isolated. Three of the isolates described here were identified as *Arthrobacter*, two as *Bacillus* and two as *Mycobacterium* species (Table 1). These were selected for genomic sequencing out of multiple isolates, and each isolate in the same phylogenetic affiliation originated from a different enrichment microcosm. The *Arthrobacter* and the *Bacillus* strains were only isolated from the enrichments set up at 10 °C or at room temperature, while the *Mycobacterium* strains were only isolated from enrichments set up at 30 °C. The *Arthrobacter* species were represented by two distinct phylotypes, forming either bright yellow (strain 31Y) or whitish colonies (strains 35W and MA-N2). The two *Bacillus* isolates and the two *Mycobacterium* isolates, respectively, were similar phenotypically. All isolates were facultative methylotrophs, able to grow on rich media, such as nutrient broth agar (Difco), and on multiple multicarbon substrates, such as succinate or glucose, as previously reported for these types [1]. However, none was able to grow on methanol.

### 3.2. The Newly-Sequenced Genomes Represent a Diversity of Gram-Positive Methylotrophs

Based on the 16S rRNA gene sequence, the closest named relative of all three *Arthrobacter* isolates was *Arthrobacter aurescens* (97.4% to 99.8% 16S rRNA gene sequence identity). However, they were only distantly related to each other (96.7% to 97.5% 16S rRNA gene sequence identity). Both white isolates were somewhat more closely related to the yellow isolate than to each other.

The two *Mycobacterium* isolates were closely related to each other (99.4%) and closely related to the differently named species of *Mycobacterium* (*M. rhodesiae*, *M. gilvum*, *M. smegmatis*; 97.6% to 97.8%).

The two *Bacillus* isolates were closely related to each other (100% 16S rRNA gene sequence identity), but not to the described *Bacillus* species, including the well-studied *B. methanolicus* species strains, MGA3 and PB1, with which they only showed 93.0% and 93.1% 16S rRNA gene identity, respectively.

**Figure 1:**
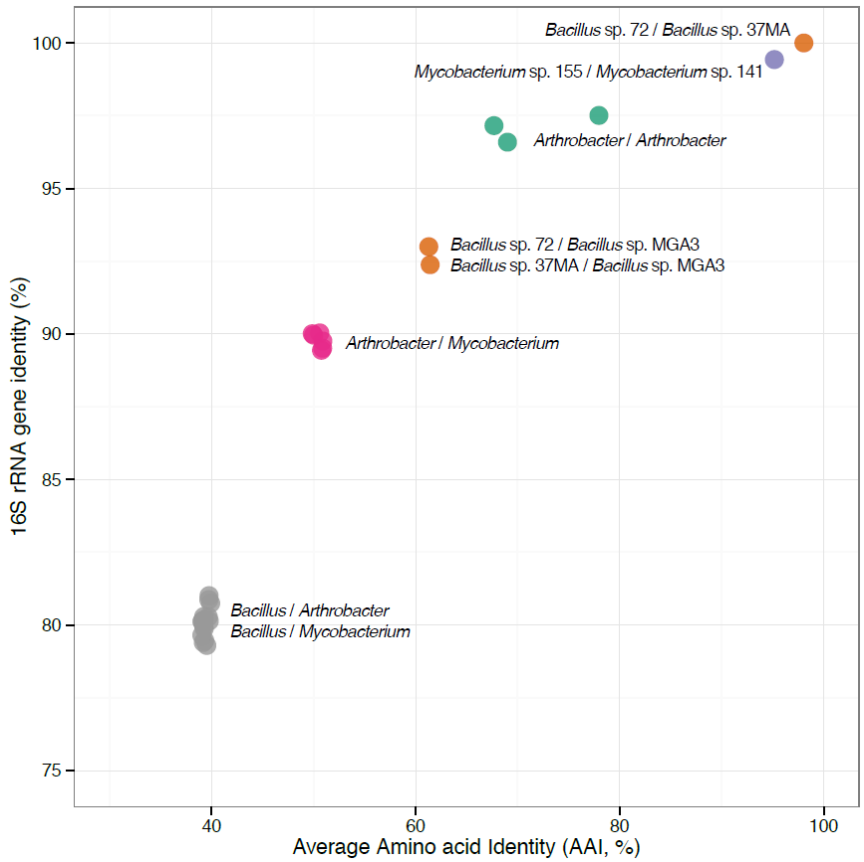
Phylogenetic relationships among the strains described. Each dot represents a comparison between two genomes and shows their 16S rRNA gene identity (*y*-axis) plotted against the average amino acid identity (AAI) of the genes shared between the two genomes (*x*-axis).

We further assessed the genomic diversity of the isolates in terms of genome-genome similarity, via calculating average amino acid identity indices (AAI) [16,17]. As expected, the two *Mycobacterium* genomes and the two *Bacillus* genomes were found to be very similar in terms of protein-protein identity (95% and 98% AAI, respectively; Figure 1). However, the proteins translated from the *Bacillus* genomes shared only 61.2% AAI with the proteome of *B. methanolicus*. The *Bacillus* (low GC Gram-positive organisms) proteomes shared only 39% AAI with the *Arthrobacter* or the *Mycobacterium* (high GC Gram-positive organisms) proteomes. Within the high GC Gram-positive group, significant divergence was uncovered, with the three *Arthrobacter* species only sharing 68% to 78% AAI and the *Mycobacterium* genomes sharing only 50% AAI with the *Arthrobacter* genomes (Figure 1).

### 3.3. Methylotrophy Pathways Deduced from the Novel Genomes Agree with Prior Knowledge, but Suggest Novel Primary Oxidation Modules

Methylotrophy pathways so far have been only thoroughly analyzed at the systems level in one representative of Gram-positive methylotrophs, *B. methanolicus*. The methylotrophic metabolic scheme in this bacterium [13,14,15], as well as the individual methylotrophy genes previously identified via functional studies in a variety of Gram-positive methylotrophs [9,10,12,18] served as guides in reconstructing methylotrophy metabolic modules in the new genomes.

#### 3.3.1. Primary C1 Oxidation Modules

None of the organisms included in this study were able to grow on methanol. Thus, not surprisingly, no genes for methanol oxidation were identified in any of the genomes, by using protein queries for the NAD-dependent methanol dehydrogenase from *B. methanolicus* [13,15] or the *N*,*N*-dimethyl-4-nitrosoamiline oxidoreductase from *Mycobacterium* sp. [12].

Queries for methylated amine oxidation modules produced the following results. Genes for trimethylamine monooxygenase (Tmo) [18] were identifiable in the genomes of *Arthrobacter* (two copies in strain MA-N2) and *Mycobacterium* strains, but not in the *Bacillus* genomes (Table 2). The *Arthrobacter* counterparts shared approximately 90% amino acid identities (AI), while *Arthrobacter*/*Mycobacterium* AI values were approximately 87%, which is significantly above the AAI values (Figure 1). The identities with proteobacterial counterparts, including the characterized proteins from alphaproteobacteria [18], were approximately 60%. Likely, the same proteins are responsible for the dimethylamine monooxygenase activity, as no additional candidates for this activity were identifiable.

**Figure 2:**
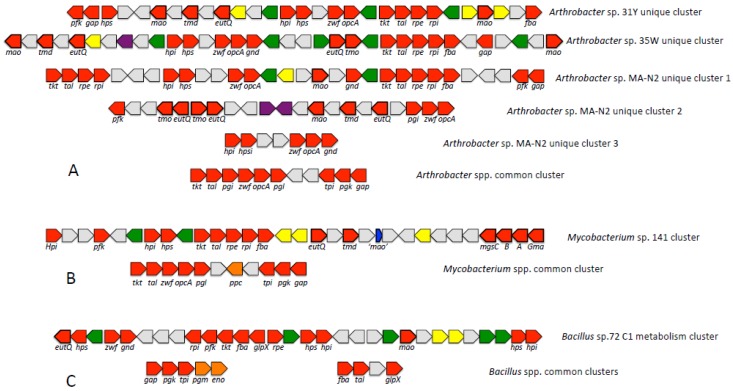
Methylotrophy gene clusters identified in the genomes described. Clusters identified in *Mycobacterium* sp. 155 and *Bacillus* sp. 37A are syntenic to the clusters shown for *Mycobacterium* sp. 141 and *Bacillus* sp. 72, respectively. In red, genes for methylotrophy functions. Genes for primary oxidation have bold lines. In green, genes encoding regulatory functions. In yellow, genes encoding transport functions. In grey, hypothetical genes or non-methylotrophy genes. In purple, genes encoding transposases. In orange, genes not essential for methylotrophy, but involved in other pathways sharing reactions with the RuMP cycle. In blue, the truncated Mao gene in the *Mycobacterium* methylotrophy clusters. Gene designations as indicated in the text. Common clusters (*i.e*., present in all strains of this genus) are identifiable not only in the strains described here, but also in multiple genomes of, respectively, *Arthrobacter*, *Mycobacterium* and *Bacillus* species. These are likely not methylotrophy specific. Genes in the unique clusters (*i.e*., unique to each strain) of *Arthrobacter* are typically highly related to the genes in the unique clusters of *Mycobacterium* (AI > 80%), suggesting lateral transfers; genes in the common clusters are less related (AI 50%–60%, in agreement with AAI), suggesting vertical inheritance.

Genes encoding methylamine oxidases (Mao) were identified via comparisons with the sequence of the biochemically characterized Mao from *Arthrobacter* P1 [10,11]. *Arthrobacter* strains 31Y and 35W each encoded two nearly-identical copies, both parts of an extended cluster encoding other methylotrophy functions (Figure 2). In the genome of strain MA-N2, two full-length gene copies are identifiable and a number of partial genes. As this is a draft sequence, some of the incomplete copies may be the result of poor assembly. Alternatively, they may be the result of genomic evolution. The *Bacillus* genomes encode three nearly-identical copies each, with approximately AI 45% with the *Arthrobacter* counterparts.

**Table 2:** Enzymes involved in methylotrophy.

	*Arthrobacter* sp. 31Y	*Arthrobacter* sp. 35W	*Arthrobacter* sp. M-N2	*Bacillus* sp. 37MA	*Bacillus* sp. 72	*Mycobacterium* sp. 141	*Mycobacterium* sp. 155
Tmo	1611	3598	1313, 1316	-	-	2303	3391
Tmd	2725	3579	1325	-	-	2887	3922
Mao	2723, 2743	3577, 3611	1074, 1322/1323, 3581, 4240, 4249	1646, 4043, 4264/4265	0038, 0528, 3848	2885 (partial)	3920 (partial)
EutQ *	1610, 1786, 2727, 2952	1944, 3581, 3597	1314, 1315, 1327	2690, 2768, 4276, 4302	0506, 0538	2304, 2889	1719, 3392, 3929
Gma	-	-	-	-	-	2874	3910
MgsA	-	-	-	-	-	2875	3911
MgsB	-	-	-	-	-	2876	3912
MgsC	-	-	-	-	-	2877	3913
MgdA	2010	-	1962	-	-	1509	2645
MgdB	2011	-	1963	-	-	1510	2646
MgdC	2012	-	1964	-	-	1511	2647
MgdD	2013	-	1965	-	-	1512	2648
FolD1	3659	0111	2114	2264	1211	1451	2593
FolD2	2859	-	-	-	-	-	-
Mch	2860	-	-	-	-	-	-
PurU1	3656	0108	2111	3324	2413	1098, 2609	2235, 3667
PurU2	2015	-	1967	-	-	-	-
PurU3	2862	-	-	-	-	-	-
Fhs	0972	-	0487	-	-	-	-
FaDH	2857	-	-	-	-	2016	-
Fdh1A	-	-	3202	-	-	1890	3030/3031
Fdh1B	-	-	3201	-	-	1889	3029
dh1C	-	-	3200	-	-	1888	3028
Fdh2	-	-	-	3013	1944	-	-
Fdh3	3415	-	1898	-	-	-	-
Fdh4	2858	0024	-	-	-	-	-
Fdh5	-	-	3418	-	-	-	-
Pgi1	4707	2121	1329	-	-	-	-
Pgi2	-	-	-	2633	1591	-	-
Pgi3	-	-	-	-	-	1659, 1660	2797, 2798
Zwf1	2734, 4706	1501, 3590, -4201	1079, 1690	-	-	0485	1591
Zwf2	0544	2030	0009	-	-	-	-
Zwf3	-	-	-	-	-	0102, 3386	0013
Zwf4	-	-	-	2231	1177	-	-
Zwf5	-	-	-	4315	0509	-	-
OpcA *	2735, 4705	1502, 3591, 4202	1078, 1330/1331, 1691, 3346, 3572/3573	-	-	0486	1592
Pgl	4704	1503	3347, 3572	0487	1593	3368, 3220	2456,2308
Gnd1	0504	1989, 3592	1692, 3535	2234, 3220	1184, 2308		
Gnd2	2377	3418	1072, 4056	-	-	0096, 2519	3586
Gnd3	-	-	-	4314	0510	-	-
Hps	2720, 2732	2137, 3588	1082, 1667, 1687	2007, 2010, 4272/4273, 4303, 4317	0507, 0521, 0535, 2219, 2222	2900	3939
Hpi	2731	2136, 3587	1083, 1668, 1686	4274	0522, 0536	3940, 3946	2901, 2907
Pfk1	1057, 2718	2691, 3605	0573, 1061, 4242, 4265	4309	0515	2904	3943
Pfk2	-	-	-	2525	1481	0976	2045
Pfk3	4492	-	-	-	-	-	-
Fba	2746, 2929	3604, 3822	1066, 1250	3273, 4307	0517, 2360	1980, 2894	3125, 3933
Tkt	2737, 4709	1498, 3600	1070, 1091, 3342	1128, 4308	0372, 0576	0483, 2898	1589, 3937
Tal	2738, 4708	1499, 3601	1069, 1090, 3343	3279	2359	0484, 2897	1590, 3936
GlpX1	3729	0180	2184	3270	2355	3603	0231
GlpX2	-	-	-	3354, 4306	0578, 2442	-	-
Rpe	2739, 4222	0804, 3602	1068, 1089, 2663	4305	0519	2896	3935
Rpi1	0371, 2740	1824, 3603	1067, 1088, 3504	4310	0514	2895	3934
Rpi2	2802, 3510	1140	0961	3357	2445	-	-
Tpi1	4701	1505	3370	2917	1868	0491	1597
Tpi2	2801	1141	-	-	-	-	-
Tpi3	3515	-	-	-	-	-	-
Gap1	4699	1507	3352	2512, 2919	1468, 1870	0493	1599
Gap2	0418, 2719	1975, 3607	1060, 3526, 4241	-	-	-	-
Pgk	4700	1506	3351	2918	1869	0492	1598
Eda	2236	-	4368, 4405	-	-	-	-

For enzyme abbreviations, see the text. Numbers correspond to gene numbering in each genome as annotated in the Integrated Microbial Genomes (IMG)/M database (http://img.jgi.doe.gov/cgi-bin/w/main.cgi). * EutQ, protein of unknown function that we propose to be involved in methylated amine oxidation, based on its conspicuous location near genes for Mao, Tmo and Tmd; OpcA, protein essential for the activity of Zwf in some organisms [33].

Remarkably, only small fragments of the *mao* gene are identifiable in the genomes of the *Mycobacterium* strains, as parts of conserved genome islands containing other methylotrophy genes (Figure 2). As the sizes of the truncated genes are very similar (129 and 123 nucleotides, respectively), it seems likely that these genes are remnants of a deletion event. The scenario in which two independent assemblies resulted in these truncated genes is less likely. If no functional Mao is encoded in the *Mycobacterium* genomes, how then is methylamine oxidized by these species? We hypothesize that it is oxidized via the *N*-methylglutamate pathway, which is widespread among diverse microbial taxa [34,35,36]. In the vicinity of the truncated *mao* genes, in each genome, we identified a cluster of genes similar to the genes encoding gamma-glutamylmethylamide synthetase (Gma) and the three subunits of *N*-methylglutamate synthase, well-characterized in Proteobacteria (MgsA–C) [34,35]. No such gene clusters are present in other genomes included in this study. Gene clusters were also identified in the *Mycobacterium* genomes potentially responsible for the *N*-methylglutamate dehydrogenase function (MgdA–D; Table 2), also essential for the *N*-methylglutamate pathway. *mgdA–D* gene homologs at approximately 50% AI were identified in *Arthrobacter strains* 31Y and MA-N2, but not in the remaining genomes (Table 2).

The product of trimethylamine oxidation by the monooxygenase is trimethylamine *N*-oxide, which is cleaved into dimethylamine and formaldehyde by trimethylamine *N*-oxide demethylase (Tmd; [1,18]. While this enzyme has been partially purified from a number of sources, including methylotrophic bacteria [19,37], the sequence of the protein responsible for this activity remained unknown. Recently, it has been proposed that an aminomethyltransferase carries out this function in Alphaproteobacteria, in whose genomes the respective gene is co-located with the gene for Tmo [38]. In the genomes characterized here, we were unable to identify such a gene in the vicinity of the Tmo genes. Instead, we observed the presence of a conspicuous gene encoding a protein predicted to be a ferredoxin and a flavodoxin, possessing a NAD(P)-binding motif. These predicted properties match the properties of the characterized Tmd enzymes [1,19,37], suggesting this gene as a candidate for encoding the Tmd. Clearly, this proposed function requires experimental verification. Other conspicuous genes were present in the vicinity of putative methylated amine oxidation genes, annotated as ethanolamine utilization genes EutQ, whose function so far remains unknown. These encode small proteins of the cupin family. In each case, almost identical copies of EutQ are found near Tmo and Mao genes (Figure 2), and these are highly related (>80% AI) among the *Arthrobacter* and the *Mycobacterium* species. EutQ-like genes are also found in the C1 gene cluster in *Bacillus*, but they are not related to the genes in *Arthrobacter* or *Mycobacterium*. We hypothesize that the *eutQ* genes encode proteins important for methylated amine oxidation, thus we included these proteins in our gene inventory analysis (Table 2, Figure 2).

#### 3.3.2. Potential for Oxidation of Formaldehyde to CO_2_ via Linear Pathways

Gram-positive methylotrophs have been suggested to utilize the cyclic pathway for formaldehyde oxidation to CO_2_, based on high activities of the respective enzymes [1,18]. However, experimental evidence was obtained for *B. methanolicus* that a linear pathway is also active [15]. We were able to identify genes for the relevant H_4_F-linked C1 transfers in all of the genomes. With the exception of strain 31Y, a single FolD (bifunctional methylene-H_4_F dehydrogenase/methenyl-H_4_F cyclohydrolase) was encoded, and their reciprocal AI fit well within the AAI ranges (Figure 1). The genome of strain 31Y encoded a second FolD, with only 32% AI with its homolog. The latter gene was part of a unique gene cluster encoding C1 transfer reactions, including an additional, non-homologous methenyl-H_4_F cyclohydrolase (Mch), PurU (formyl-H_4_F deformylase), a formaldehyde dehydrogenase (NAD-independent), an aminomethyltransferase and a molybdenum oxidoreductase of unknown function (genes 2855–2863). Only one other genome encoded a recognizable formaldehyde dehydrogenase (Fadh), the genome of *Mycobacterium* sp. 141, with 78% AI to its counterpart in *Arthrobacter* sp. 31Y. Only the genomes of *Arthrobacter* strains 31Y and MA-N2 encoded formyl-H_4_F ligases similar to the one in the genome of *B. methanolicus* (Fhs) [13,15]. However, all genomes encoded PurU enzymes: a single copy in the *Bacillus* genomes, two copies (similar, but not identical) in the *Mycobacterium* genomes and one to three relatively divergent copies in the *Arthrobacter* genomes. The prevalence of PurU (an irreversible enzyme) over Fhs (FtfL, a reversible enzyme) [39] indeed supports a proposal for the dissimilatory function of the H_4_F-linked pathway [15].

Formate dehydrogenases (Fdh) are encoded by all genomes, and they represent a variety of phylogenetically distant types. The genomes of the *Mycobacterium* species encode a three-subunit type (Table 2). Only one *Arthrobacter* strain, MA-N2, encodes a homolog of this enzyme (AI in the range of AAI). The genomes of the *Bacillus* species encode a single-subunit enzyme (Fdh2) highly similar to the one annotated in *B. methanolicus* [13,15], but without homologs in either *Arthrobacter* or *Mycobacterium*. The *Arthrobacter* species encode three other types unrelated to the first two, as follows. Strains 31Y and MA-N2 encode Fdh3 (AI 90%); strains 31Y and 35W encode an Fdh4 (AI 73%); and MA-N2 encodes Fdh5 that is equally distantly related to Fdh3 and Fdh4 (AI 52%–58%) and unrelated to Fdh1 and Fdh2.

#### 3.3.3. Oxidation of Formaldehyde to CO_2_ by the Cyclic Pathway

Gram-positive methylotrophs are known to use the dissimilatory RuMP cycle for formaldehyde oxidation to CO_2_ [1,37]. This cycle shares the early reactions, catalyzed by hexulose phosphate synthase (Hps) and hexulose phosphate isomerase (Hpi), that produce fructose 6-phosphate, the genes for which will be described below. Genes for glucose phosphate isomerase (Pgi) were identified in all genomes, with two nearly-identical copies present in the *Mycobacterium* strains. Interestingly, the proteins encoded by the *Arthrobacter* strains were unrelated to the mycobacterial counterparts, with AI (<30%) being significantly below AAI. The *Bacillus* enzymes were unrelated to the counterparts from both *Mycobacterium* and *Arthrobacter* (AI < 29%).

Glucose 6-phosphate dehydrogenases (Zwf) are also represented by a variety of phylogenetic types, with multiple copies present in each genome (Table 1). The Actinobacteria share one type, with AI 70% between *Arthrobacter* and *Mycobacterium* species (above AAI). The remaining types are specific to individual groups with low AI among the groups (<40%). The genomes encoded one to two 6-phosphogluconolactonases. The *Arthrobacter* counterparts were only distantly related to the mycobacterial counterparts (AI 46%–47%), and none shared any identity with the *Bacillus* counterparts.

The phylogenetic landscape of the enzyme concluding the oxidative cycle, 6-phosphogluconate dehydrogenase (Gnd), was also complex, with three recognizable types being encoded. All three groups appeared to share one type, with AI values slightly above AAI. The second type was only shared among the Actinobacteria, again with AI being above AAI. The third type was exclusive to *Bacillus*.

#### 3.3.4. The Assimilatory RuMP Cycle

Multiple copies of the first enzyme of the RuMP cycle, Hps, were identified in all genomes, in each case identical or nearly identical to each other within each genome. The actinobacterial genomes contained two copies each, and the counterparts from *Mycobacterium* and *Arthrobacter* revealed AI (87% to 91%) that was significantly higher than AAI, suggesting that these enzymes were likely shared via lateral transfers among Actinobacteria. The *Bacillus* genomes each contained five copies of Hps genes, and these revealed low AI (41%–43%) with the actinobacterial counterparts, in agreement with AAI. One to three genes were identified in each genome encoding Hpi, and the trend for relative evolutionary distances was repeated: the actinobacterial counterparts were more related to each other than expected from AAI (74%–77%), while the *Bacillus* counterparts were related to the actinobacterial counterparts at the AAI value. Multiple phosphofructokinases are also encoded. While in the *Arthrobacter* species, most copies are highly related (the exception is gene 4492 in *Arthrobacter* 31Y), the *Mycobacterium* species encode two dissimilar enzymes. One shares unusually high AI (90%) with *Arthrobacter*, suggesting a lateral transfer event, while the second is shared among the species at AI equaling AAI, suggesting vertical inheritance. Two distinct types are present in *Bacillus* (60% AI). Multiple copies of fructose bisphosphate aldolases (Fba) were present, of which one was shared by *Arthrobacter* and *Mycobacterium* species at AI > 80%, the other being more divergent between these species. The *Bacillus* counterparts were unrelated to the actinobacterial counterparts. Similar trends were observed for many of the enzymes that participate in regeneration of the acceptor molecule as part of the RuMP cycle [1]. Of the multiple copies of transketolase (Tkt) transaldolase (Tal), ribulose phosphate epimerase (Rpe) and ribulose phosphate isomerase (Rpi), fructose 1,6-sedoheptulose 1,7-bisphosphatase (GlpX), triosephosphate isomerase (Tpi), glyceraldehyde phosphate dehydrogenase (Gap) and phosphoglycerate kinase (Pgk), some copies revealed higher than expected AI between the *Arthrobacter* and *Mycobacterium* counterparts, while others were not phylogenetically related, suggesting complex histories.

To test for the potential for the alternative variant of the RuMP cycle, we queried the genomes for the presence of genes potentially encoding phosphogluconate dehydratase and 2-keto-3-deoxy-6-phosphogluconate aldolase (Eda) [1]. Gene homologs for the latter were only detected in *Arthrobacter* strains 31Y and MA-N2, but gene homologs for the former were not detectable in any of the genomes.

### 3.4. Analysis of Distinct C1 Gene Clusters Suggests a Means for the Evolution of Methylotrophy in Gram-Positive Methylotrophs

In all of the genomes analyzed here, many of the genes implicated in methylotrophy were found as parts of genomic C1 metabolism islands (Figure 2; note that as these are draft genomes, the islands as shown may be incomplete, and some of the singleton genes may actually belong to islands). These islands in most cases contain genes for both primary C1 oxidation and the downstream functions that include oxidation and assimilation of formaldehyde. Clustering of C1 genes is also typical of other methylotrophs, including Proteobacteria, Verrucomicrobia and NC10 phylum species [39]. In cases where the regulation of gene or protein expression was studied, coordinated regulation was noted for genes/proteins involved in specific branches of C1 metabolic pathways [15,40], and co-transcription is a common mechanism [41]. Thus, one strategy for evolving methylotrophy seems to be clustering of relevant genes on the chromosomes. The other strategy must be multiplication of genes for key and likely rate-limiting functions, such as primary C1 oxidation and the early steps of C1 assimilation. Multiple and nearly identical copies were identified for these functions in most of the genomes analyzed here. Lateral transfers of gene clusters among diverse Gram-positive methylotrophs present another prominent mechanism. The proteins encoded by the specific C1 gene clusters in the *Arthrobacter* and the *Mycobacterium* species (Figure 2) display significantly higher relatedness than predicted from whole genome comparisons (Figure 1), strongly suggesting that these species either exchanged these clusters or acquired them from the same donor. However, an argument for the vertical evolution of methylotrophy in Gram-positive bacteria is also valid, as proteins in C1 pathways are only distantly related between the *Bacillaceae* and Actinobacteria, while the biochemical schemes are essentially identical.

How many of the *Arthrobacter*, *Bacillus* and *Mycobacterium* species are methylotrophic? Can we predict this capability from looking at the constantly growing genomic databases? Indeed, BLAST analyses identify homologs for most of the genes encoding functions necessary for methylotrophy (Table 2) in multiple sequenced genomes. However, no predictions could be made based on single genes. For example, even the “signature” functions, such as Hps and Hpi, are not necessarily indicative of methylotrophy, as they are also present in the genomes of non-methylotrophs [38,42]. The situation is further complicated by the fact that most of the reactions involved in assimilation or dissimilation of formaldehyde, including C1 transfer reactions or sugar phosphate inter-conversions, are common to all life. Thus, complete sets need to be identified in the genomes, with special attention to gene clustering. The genomes whose sequencing we are reporting here present some of the first blueprints for such analyses, in addition to the genomes of *B. methanolicus* [13,14,15].

## 4. Conclusions

The genomic sequences of seven novel strains representing the genera of *Arthrobacter*, *Bacillus* and *Mycobacterium*, all methylated amine utilizers, present an important reference dataset for understanding the genomic basis of methylotrophy in Gram-positive methylotrophic bacteria. This study is a major contribution to the field of methylotrophy, aimed at closing the gap in the genomic knowledge of C1 metabolism within this diverse group of bacteria.
